# Single variable domains from the T cell receptor β chain function as mono- and bifunctional CARs and TCRs

**DOI:** 10.1038/s41598-019-53756-4

**Published:** 2019-11-21

**Authors:** Julyun Oh, Dora Toledo Warshaviak, Mikayel Mkrtichyan, Melanie Lisette Munguia, Abby Lin, Falene Chai, Craig Pigott, Jaspal Kang, Michael Gallo, Alexander Kamb

**Affiliations:** 1 A2 Biotherapeutics, Inc. 30301 Agoura Rd., Agoura Hills, CA 91301 USA; 2 Innovative Targeting Solutions, Inc. 290-2985 Virtual Way, Vancouver, BC V5M 4X7 Canada

**Keywords:** Applied immunology, Cancer immunotherapy

## Abstract

Cell therapy using T cell receptors (TCRs) and chimeric antigen receptors (CARs) represents a new wave of immunotherapies garnering considerable attention and investment. Further progress in this area of medicine depends in part on improving the functional capabilities of the engineered components, while maintaining the overall size of recombinant constructs to ensure their compatibility with existing gene delivery vehicles. We describe a single-variable-domain TCR (svd TCR) that utilizes only the variable domain of the β chain (Vβ). This Vβ module not only works in TCR and CAR formats, but also can be used to create single-chain bispecific CARs and TCRs. Comparison of individual ligand-binding Vβ domains in different formats suggests that the lone Vβ sequence controls the sensitivity and a major part of the specificity of the CAR or TCR construct, regardless of signaling format, in Jurkat and primary T cells.

## Introduction

Cell therapy promises to revolutionize certain aspects of medicine, and has recently achieved a significant milestone with approval of the first engineered chimeric antigen receptor T cells (CAR-Ts)^1,2^. This therapeutic modality depends on receptor molecules, typically engineered antibody single-chain variable fragment (scFv) domains, to redirect T cell activity to specific cell surface target antigens expressed on the cancer cell. Because recurrently expressed, tumor-selective surface proteins are extremely rare among solid tumors, drug discovery has recently begun to explore the utility of peptide-MHC complexes (pMHCs) as target opportunities for CAR-Ts and T-cells with engineered receptors or native TCRs.

pMHCs are the natural ligands for TCRs and as such lend themselves to targeting by TCRs and their derivatives^3^. Class I MHC molecules consist of a molecular dimer of an α chain associated with β2 microglobulin. This dimer binds in a degenerate manner to individual short peptides while folding in the endoplasmic reticulum to create the pMHC complex^4^. The pMHC is then transported to the membrane surface where it can be recognized by cognate T cells. If a certain binding threshold is crossed, pMHCs can trigger activation and effector function of T cells through TCRs.

T cell receptor heterodimers (αβ) are expressed on T cell surface in complex with the CD3 proteins, which provide the necessary signaling domains in the stoichiometry α: β: γ: δ: 2ε: 2ζ ^5^. The α and β subunits form the central pMHC binding element, which has significant structural similarity to its relative, the antibody antigen-binding fragment (Fab). Variable domains of the α and β subunits are assembled from discrete V, D, and J gene elements via genomic recombination. The β chains are inherently more complex than the α chains since they include D regions and two junctional regions (V-D and D-J), whereas α chains just have one (V-J). Both the α and β subunits typically contribute to binding a small region on the class I MHC ligand distal to the membrane that includes residues from the MHC molecule as well as the short peptide complexed with it^6,7^.

pMHCs afford the option to target peptides derived from intracellular proteins that reside in the cytoplasm and are otherwise inaccessible to large molecules, such as monoclonal antibodies. As more elaborate cell therapeutics are contemplated, designed and deployed, the small size and modularity of the component parts become increasingly valuable. For example, the creation of bifunctional receptors, i.e., receptors with two linked domains that bind different ligands, affords the option to target two different antigens at once, a potentially very valuable asset in certain therapeutic settings. However, some aspects of TCRs may limit their utility as modular components of large-molecule and cell-therapy applications, constraints potentially rooted in their basic structure and function. Such structural complexity and increased size may hamper the practical development of next-generation bifunctional therapeutics.

The appeal of bifunctional targeting molecules, coupled with packaging limitations of gene-transfer vectors such as lenti- and retroviruses, raises the question of whether TCRs can function as a single binding domain; e.g., as a Vβ devoid of its normal partner, the α variable domain (Vα). To our knowledge, no instance of such a streamlined TCR has been reported, though shark species have a variety of novel TCRs that are distinct from mammalian orthologs^8,9^. If such a simplified TCR variable domain that retains specificity and functional activity against particular pMHCs were possible, it could open a path to engineering smaller, more complex binding capabilities with svd TCRs.

Though svd TCRs are an attractive concept, it is not evident that such stripped-down binding domains can be derived easily from mammalian TCRs. The three-dimensional structure of TCRs reveals a complex where the TCR Vα and β chains pack together, covering a large (>200 Å^2^) hydrophobic surface. Removal of the Vα domain exposes this surface to destabilizing solvent interactions. By analogy with protein engineering efforts on antibodies conducted over the past decades, such dramatic changes in structure require significant adjustments, either by design or selection, to accommodate the single-variable domain format^10,11^. Despite the challenges, antibody VH-only domains are increasingly popular for generation of innovative clinical molecules with multi-specific binding properties^12,13^.

Here we demonstrate that svd TCRs can be constructed from a Vβ domain with no additional sequence engineering within the framework and constant regions of the β chain. These svd TCRs express stably on the surface of mammalian cells, including T cells. They bind pMHC tetramers selectively and appear to trigger T cells in much the same manner as full TCRs. Moreover, they function in tandem as CAR and TCR bifunctional proteins. Such Vβ-only domains provide a powerful basis for next-generation engineered T cell therapeutics and shed light on the mechanisms that dictate signaling sensitivity of TCRs and CARs.

## Results

### Identification of β-only pMHC binding domains

To identify peptide-specific pMHC-binding molecules, V(D)J recombination in cell culture was used to generate sequence diversity in the TCR Vβ domain while keeping the Vα constant in a controlled fashion in HEK293 cells (see patent WO2017/091905 for details about the HuTARG^TM^ platform used to generate the TCR diversity). Over 100 million TCR-expressing cells were sorted for TCRs specific to the HLA-A*02-01 allele in complex with NY-ESO-1 9 V variant peptide (SLLMWITQV) or MAGE-A3 peptide (FLWGPRALV) using pMHC multivalent tetramers. Note that C-terminal to the Cα/β domains, the library construct encoded the human CD3ζ transmembrane and cytoplasmic domains fused to the β chain to facilitate its expression in HEK293 cells, and subsequent analysis. Specific pMHC-binding cells were enriched over multiple rounds and purified from the library. During the process, α chain expression was inadvertently lost and β chains that endow specific epitope-binding in the absence of a second TCR variable domain were recovered. Such β-only clones were used as the basis of the studies described below.

### β-only domains express stably and show selective pMHC binding in CAR and TCR formats

Several clones of β-only domains selective for either HLA-A2/NY-ESO-1 peptide or HLA-A2/MAGE-A3 peptide were recovered from the HuTARG™ sort and sequenced. Among these, 3 and 4 unique CDR3 sequences were identified for NY-ESO-1- and MAGE-A3-selective binders, respectively (Supplementary Table S1). Interestingly, all 7 β idiotypes for both pMHC targets utilize the TRBV5–8*01 segment, suggesting that there are structural properties of this Vβ segment that facilitate stability of a single domain in the absence of its normal Vα partner (see below). Furthermore, the average CDR3 loop length was 16.3 amino acids (range 12–20), ~2 amino acids longer than the average observed among a set of human TCR Vβ sequences^14^. These idiotypes were characterized for their ability to express in HEK293T cells and bind pMHC targets by flow cytometry. The TCR βF1 (8A3) antibody was used to detect surface-expressing TCRβ chains. This antibody does not stain the native form of the TCR^15^, allowing detection of the TCRβ chain in CAR formats only. pMHC tetramers were generated by conjugating biotin-labeled, peptide-containing MHC Class I A*02:01 with fluorophore-labeled streptavidin in a tetramer format. All idiotypes were expressed stably on the cell surface with various tetramer-binding abilities (Supplementary Fig. S1). The strongest binders, idiotype #2 for a NY-ESO-1-targeted Vβ-only domain and idiotype #5 for a MAGE-A3-targeted domain, were selected for further studies.

**Figure 1:**
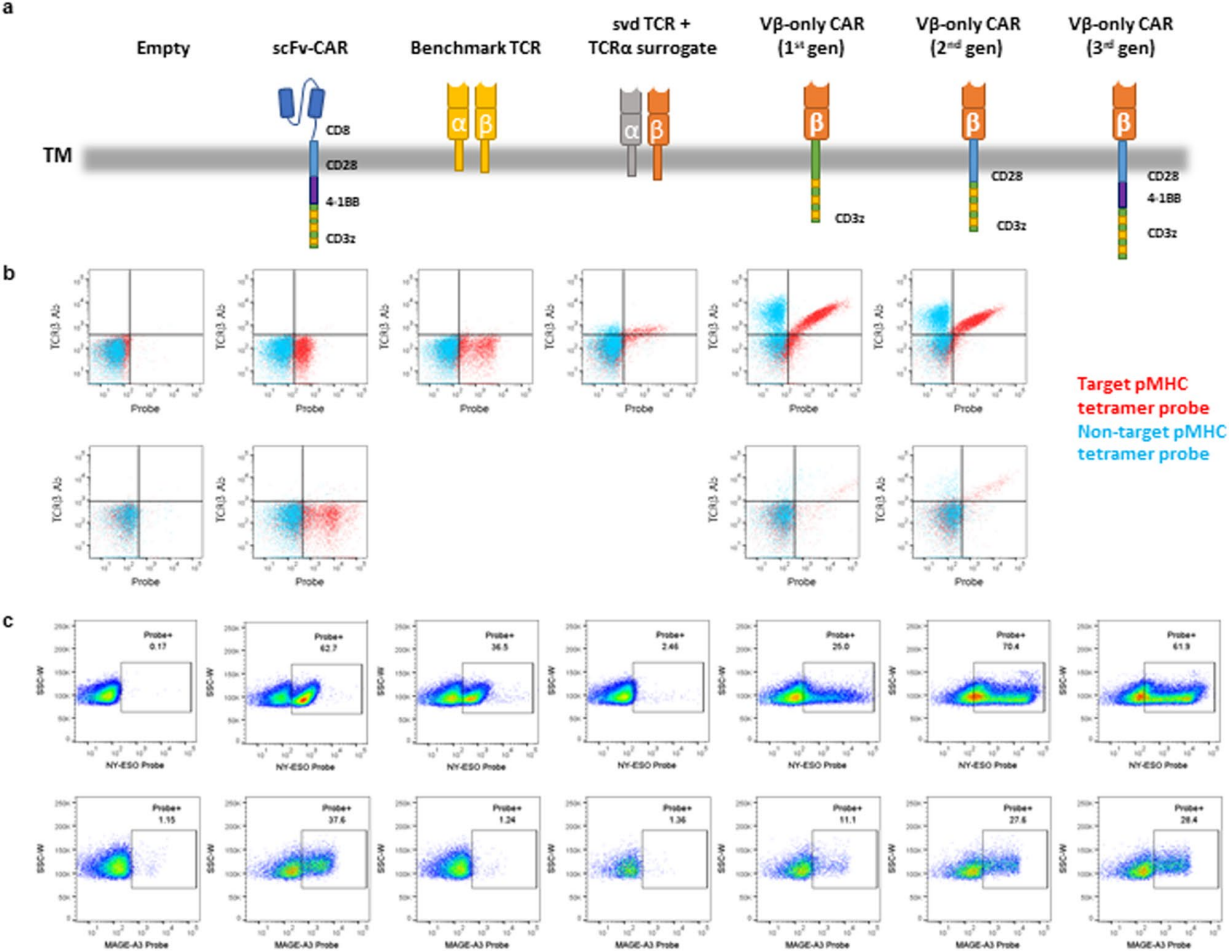
Vβ-only domains in CAR formats express stably and show selective pMHC binding. (**a**) Schematic of the constructs used in the experiments. Note that the 1^st^ gen Vβ-only CAR will dimerize through CD3ζ TM, which is not depicted in the schematics. (**b**) FACS plot of HEK293T cells transfected with NY-ESO-1 (top) and MAGE-A3 (bottom) pMHC-targeted constructs. The x-axis reflects the amount of target (red) and non-target (blue) pMHC tetramer bound to cell. The y-axis reflects the amount of the TCRβ chain on the cell surface. (**c**) FACS plot of Jurkat cells transfected with NY-ESO-1 (top) and MAGE-A3 (bottom) pMHC-targeted constructs. The x-axis reflects the amount of target pMHC tetramers bound to cell.

To assess the specificity and versatility of these Vβ-only domains, the NY-ESO-1- and MAGE-A3-binding idiotypes (#2 and #5, respectively) were formatted as TCRαβs (referred to as svd TCR) and CARs (referred to as Vβ-only CAR). For the TCRαβ format, a generic α chain that is abundantly expressed in humans was used as a surrogate (TRAV41*01/J49*01). For the CAR format, Vβs were linked to the β chain constant region (Cβ), which in turn was fused to a CD3ζ transmembrane and intracellular signaling domain (first-generation CAR) or a CD28 transmembrane region and CD28 and CD3ζ intracellular signaling domains (second-generation CAR) (Fig. 1a). For comparison we used the previously reported NY-ESO-1-targeted TCR clone 1G4^16^ and MAGE-A3-targeted mouse TCR^17^, as well as CARs with scFvs derived from reformatted antibody surface display (HuTARG^TM^) libraries. These constructs were transfected into HEK293T cells to assess expression and binding specificity by flow cytometry. Mixtures of 7 off-target pMHCs with different HLA allele and unrelated peptides were used to generate negative-control tetramer. Flow cytometry data showed that both NY-ESO-1- and MAGE-A3-targeted Vβ-only CARs are stably expressed and selectively bind to target tetramers in HEK293T cells. svd TCRs also express and show specificity for the pMHC target, but with significantly lower binding (Fig. 1b).

To examine expression and function of Vβ-only CARs and svd TCRs in a more natural setting, the constructs were transfected into the Jurkat human T cell line. Additional CAR constructs with the Vβ-Cβ domains linked to CD28 transmembrane region with CD28, 4-1BB and CD3ζ intracellular signaling domains (third-generation CAR) were also tested. All NY-ESO-1- and MAGE-A3-targeted Vβ-only CARs showed detectable tetramer binding in Jurkat cells. Slightly higher tetramer binding percentages were observed in second- and third-generation CARs compared to first-generation. However, svd TCR did not bind tetramer for either pMHC target (Fig. 1c).

### The TRB5–8 Vβ segment encodes a sequence that may more readily form a single functional binding domain

**Figure 2:**
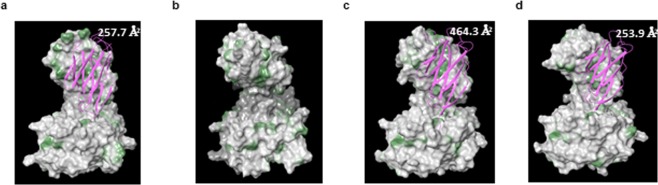
Structural homology modeling analysis. For (**a**–**d**) homology models that use the atomic coordinates of the MAGE peptide/HLA-A2 pMHC in complex with a Vβ5 TCR (5BRZ). Hydrophobic patches on the space-filling surface model are labeled green; charged and neutral surfaces white. For A, C and D, a ribbon cartoon shows where Vα normally binds to Vβ in the TCR α/β dimer. The intersection between the hydrophobic surface and the α/β contact surface was calculated and is shown in Å^2^. (**a**) MAGE-A3 svd TCR homology modeled on 5BRZ template without the TCRα subunit. (**b**) MAGE-A3 svd TCR homology modeled on 5BRZ template after 100 nsec molecular dynamics simulation. (**c**) TRBV28 domain from previously crystalized TCR (6AT6) homology model on 5BRZ template without the TCRα subunit. (**d**) TRBV9 domain from previously crystalized TCR (6AVG) homology model on 5BRZ template without the TCRα subunit.

Based on the crystal structure of a Vβ−5 TCR complexed with MAGE-A3 pMHC, we modeled one of the MAGE-A3 Vβ-only domains described here (idiotype #5) and the results matched the expectation that some adjustments to the polypeptide backbone are required (Fig. 2a)^18^. A molecular dynamics simulation of the TRB5–8 Vβ-only structure revealed that the interface with Vα is at least partly covered by a collapse of the CDR3 loop over its hydrophobic surface to accommodate the loss of Vα (Fig. 2b). In addition, modeling two other TCRs (6AT6 and 6AVG)^19^ that contain V segments from other families, suggests that at least some other Vβ domains require more radical changes to form a thermodynamically stable structure absent the Vα domain, compared to Vβ5−8 (Fig. 2c,d). Specifically, the crystal structure of a TRBV28-encoded domain shows nearly 80% more hydrophobic surface area exposed, compared to the Vβ5–8-only domain. Presumably such sequences have a more challenging route to formation of a stable single globular domain.

### Vβ-only domains in CAR formats are functionally active

**Figure 3:**
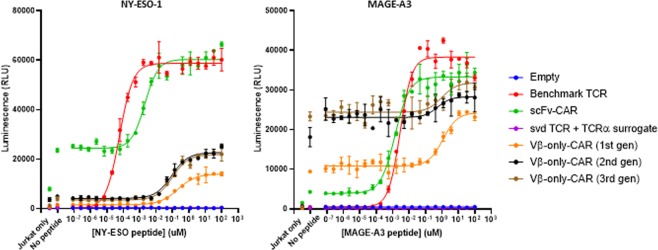
Vβ-only domains in CAR formats show target peptide-specific functional activation. NFAT-luciferase signal of transfected Jurkat cells after 6hrs of co-culture with NY-ESO-1 (left) or MAGE-A3 (right) peptide-loaded T2 cells. The error bars indicate SD (n = 2).

To assess whether target binding by Vβ-only domains can lead to functional activity, engineered Jurkat cells that expressed an NFAT-driven luciferase reporter gene were transfected with Vβ-only constructs. Serially diluted NY-ESO-1 and MAGE-A3 peptides were loaded onto T2 cells (HLA-A2+) 16 hours prior to co-culturing with transfected Jurkat effector cells. Population-averaged NFAT signal was measured by luminescence after 6 hours of co-culture. Peptide-titration curves showed that both NY-ESO-1 and MAGE-A3 Vβ-only CARs trigger peptide-concentration-dependent NFAT activation (Fig. 3). The activation is specific to its respective target peptides (Supplementary Fig. S2). First-generation CAR formats showed about two-fold less sensitivity than second- and third-generation CARs with both binders based on the EC_50_ (Supplementary Table S2). Compared to control third-generation CARs with affinity-matured scFv binders, third-generation CARs with Vβ domains were ~100 × (NY-ESO-1) and ~1000 × (MAGE-A3) less sensitive (Supplementary Table S2).

The MAGE-A3 Vβ-only CARs showed elevated background NFAT signaling (Fig. 3). However, this feature does not appear to be inherent to all Vβ-only CAR constructs as the other three idiotypes of MAGE-A3 Vβ-only modules showed lower tonic signal than the scFv CAR (Supplementary Fig. S3). To explore the cellular localization and aggregation profile of the Vβ-only CARs, transfected Jurkat cells were fixed and stained with cognate target pMHC tetramers in the presence or absence of nonionic detergent for permeabilization (Supplementary Fig. S4). No obvious difference in surface expression or intracellular aggregation among constructs with high and low backgrounds was observed.

To identify the minimal module that is required for epitope binding of Vβ-only CAR constructs, CAR vectors were generated where the Cβ domain is replaced with a CD8 hinge (Supplementary Fig. S5). Peptide-concentration-dependent NFAT signal was observed with these constructs with only modest decrease in sensitivity, indicating that Cβ is not necessary for the Vβ-only binding ability (Supplementary Fig. S5).

Cells transfected with svd TCR did not signal, consistent with the lack of tetramer binding observed in these cells.

### svd TCRs are hindered by the Vα domain

**Figure 4:**
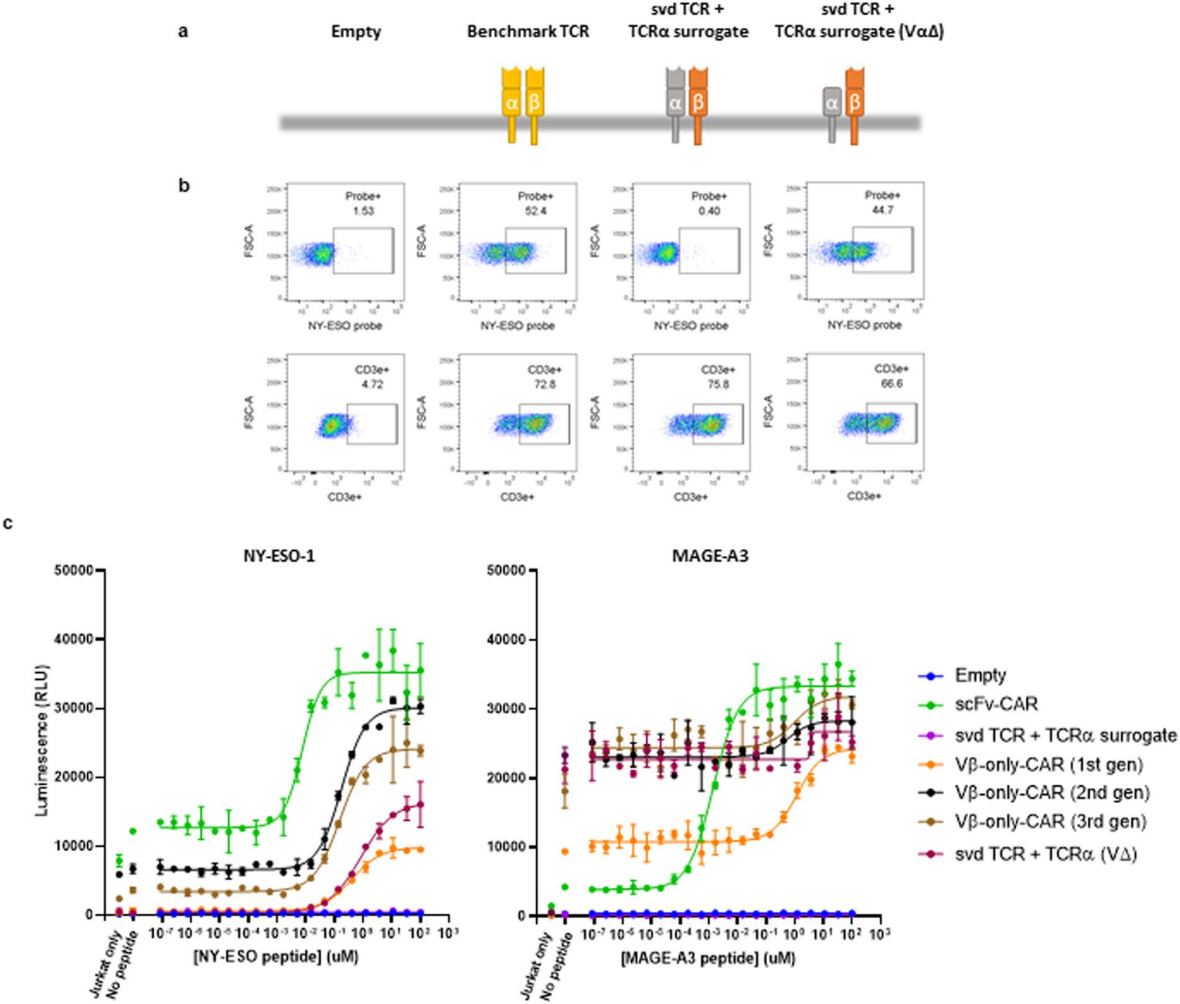
Removal of Vα enables svd TCR function. (**a**) Schematics of the constructs tested. (**b**) pMHC tetramer binding and TCR complex formation (CD3ε surface expression) in transfected SUP-T1 cells. (**c**) NFAT-luciferase signal of transfected Jurkat cells after 6hrs of co-culture with NY-ESO-1 (left) or MAGE-A3 (right) peptide-loaded T2 cells. The error bars indicate SD (n = 2).

To further examine surface expression and full TCR complex formation in T cell lines, SUP-T1 cells were transfected with the β chains containing the Vβ-only domains and a TCRα surrogate chain. SUP-T1 cells are derived from a lymphoma with a chromosomal inversion within the TCR α and β gene cluster that prevents surface expression of the endogenous TCR complex. Because TCR α and β are not expressed, other subunits of the TCR complex, namely 4 CD3 proteins, are expressed but unable to assemble on the surface^20^. Therefore, CD3Ɛ staining was used as a readout for expression and pairing of the transfected TCRα and TCRβ constructs on the cell surface. As previously reported, the NY-ESO-1 control TCR is expressed on the surface of SupT1 cells. Moreover, the Vβ-only complex also are positive for CD3Ɛ staining, suggesting that the complex integrates the β and α subunits. However, only the control TCR showed tetramer binding, consistent with the observation in transfected Jurkat cells (Fig. 4b).

Because we observed pMHC tetramer binding of Vβ-only domains in CAR formats but not as a TCR complex, we hypothesized that the TCR Vα may hinder the Vβ-only domains’ ability to bind targets. To test this hypothesis, the Vα domain of the α chain was deleted and co-expressed with Vβ chains in SUP-T1 cells. These cells showed tetramer binding, confirming that the Vα indeed hinders epitope binding of Vβ-only domains (Fig. 4a,b). Furthermore, Jurkat cells transfected with the β and Vα-deficient chains revealed T2/peptide-dependent activation, demonstrating that the svd TCRs when their normal Vα partner is removed but the remainder of the alpha chain is retained (Fig. 4c). Despite this functional activity, tetramer binding was not observed by flow cytometry in transfected Jurkat cells, suggesting that the NFAT reporter system is more sensitive than tetramer binding as we have observed previously.

Attempts to trim the Cα domain further resulted in complete loss of tetramer binding of the modified TCRs in SUP-T1 cells and corresponding loss of functional activity in Jurkat cells (Supplementary Fig. S6). This result implies that an intact Cα domain is necessary to form a proper surrogate, and a minimal α polypeptide with only a transmembrane domain is not compatible with Vβ-only domain function, probably because of inefficient TCR complex formation absent the Cα domain, likely caused in part by exposed hydrophobic surfaces at the Cα/β interface.

### Comparison of signaling sensitivities of Vβ-only domains in CAR and TCR formats

Our system permits direct comparison of the same ligand-binding domains on a CAR and TCR. Interestingly, the sensitivity of the two formats were similar, which suggests that the ligand-binding domain—its affinity and geometry—is the main determinant of sensitivity rather than the details of the signaling mechanism that differ between the CAR and TCR. This observation is consistent with studies where we grafted scFv against the same pMHC targets onto the control NY-ESO-1 TCR, modified to accept the scFvs by removing the Vα and Vβ domains. These hybrid TCRs have sensitivities in Jurkat signaling assays that are nearly identical to the CARs from which they are derived, nearly 100x less sensitive compared to the parental TCR (Supplementary Fig. S7). These observations suggest that, at least in a controlled system where NFAT activation in Jurkat cells is the measure of sensitivity, the ligand-binding domain dictates the signaling sensitivity.

### Bispecific Vβ-only domains function in CAR and TCR formats

Recent clinical trials have revealed that current adoptive T-cell therapy is susceptible to antigen escape by tumor cells^21,22^. T cells that recognize multiple antigens offer a prospective safeguard against this problem. Bispecific receptors have been explored and developed in the context of CARs^23,24^. However, TCRs with a capacity to target two different antigens have not been reported to our knowledge. Given the small and versatile binding properties of Vβ-only domains, we suspected they might function in a more complicated format as a bifunctional CAR or TCR.

**Figure 5:**
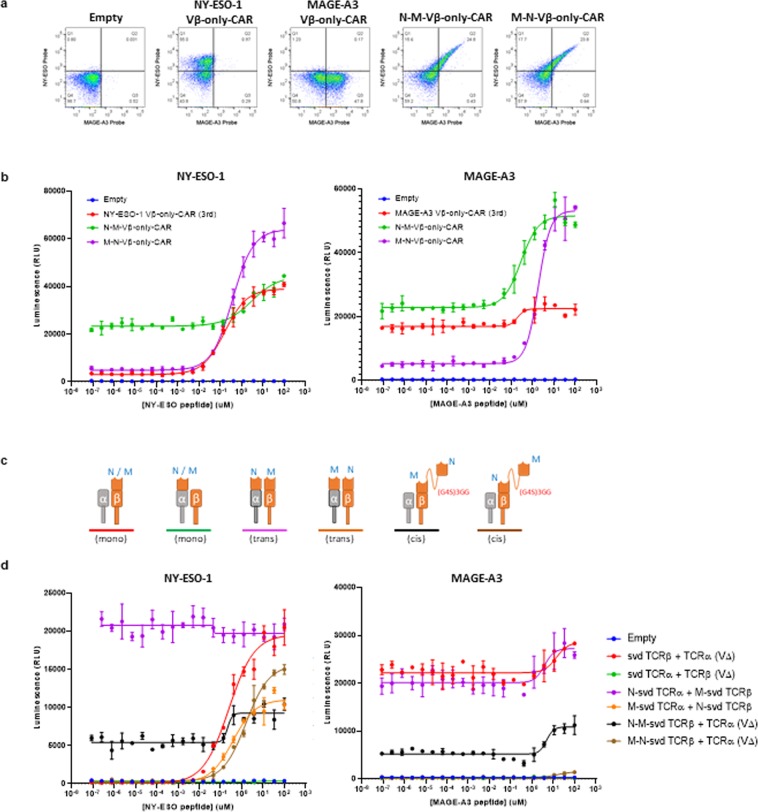
Bifunctional CARs and TCRs with two ligand binding domains. (**a**) FACS plot of transfected Jurkat cells stained for binding to NY-ESO-1 pMHC tetramer (y-axis) and MAGE-A3 pMHC tetramer (x-axis). M refers to MAGE-A3 and N refers to NY-ESO-1. (**b**) NFAT-luciferase signal of transfected Jurkat cells after 6hrs of co-culture with NY-ESO-1 (left) and MAGE-A3 (right) peptide-loaded T2 cells. The error bars indicate SD (n = 2). (**c**) Schematics of the constructs tested. M refers to MAGE-A3 and N refers to NY-ESO-1. (**d**) Same as (**b**).

To test this idea, bispecific Vβ-only CARs targeting NY-ESO-1 and MAGE-A3 pMHCs were generated in a second-generation CAR architecture with Cβ and the two Vβ domains connected in tandem via a (G_4_S)_3_GG flexible linker. Jurkat cells transfected with these constructs showed detectable binding of both NY-ESO-1 and MAGE-A3 tetramers by flow cytometry (Fig. 5a). Consistently, in the NFAT-luciferase reporter assay, bispecific CARs functioned in response to both NY-ESO-1- and MAGE-A3-peptide-loaded T2 cells with only modest declines in their individual sensitivities (Fig. 5b). The binding domain positioned further from the membrane (i.e., at the N-terminus) showed a slight decrease in sensitivity while the one directly attached to Cβ showed no change compared to the monospecific CARs.

To examine if Vβ-only domains could function as bispecific TCRs, we constructed variants in both *cis* and *trans* configurations (i.e., in tandem on the β chain, or with one Vβ domain on each chain, Cα and Cβ (Fig. 5c). As a control, Vβ fused on the TCRα chain was also expressed with a TCRβ chain that lacked a Vβ domain (i.e., Cβ only). This construct showed no function in the Jurkat NFAT-luciferase reporter assay, indicating that Vβ domains moved to the α-chain abolishes its function (Fig. 5d). Therefore, the *trans* bispecific svd TCRs showed functional activity only against the pMHC target of the binder fused on the β-chain (Fig. 5d). *Cis* bispecific svd TCRs were generated by connecting two Vβ domains in tandem via a (G_4_S)_3_GG flexible linker and expressing this construct with a surrogate TCRα chain with the Vα deleted. To our surprise, bifunctional *cis* svd TCRs with NY-ESO-1 binder on the N-terminus followed by MAGE-A3 binder N-terminal to Cβ showed both NY-ESO-1 and MAGE-A3 peptide-dependent signaling in Jurkat cells (Fig. 5d). *Cis* Vβ domains in the other orientation with the MAGE-A3 binder at the N-terminus also showed functional activity against both target peptides, although the magnitude of the signal (Emax) with MAGE-A3 peptide was reduced. The EC_50_ in assays with peptides loaded on T2 was similar for both constructs, compared to the sensitivities of monospecific parental versions of the constructs (Supplementary Table S2). We also tested if there was interaction detectable at a functional level between the two pMHC ligands when supplied to Jurkat cell expressing bispecific constructs. Nothing beyond a potentially additive effect was observed using the analytical methods of Bliss and Loewe independence^25,26^.

### Primary T cells expressing Vβ-only constructs have cytotoxic activity

**Figure 6:**
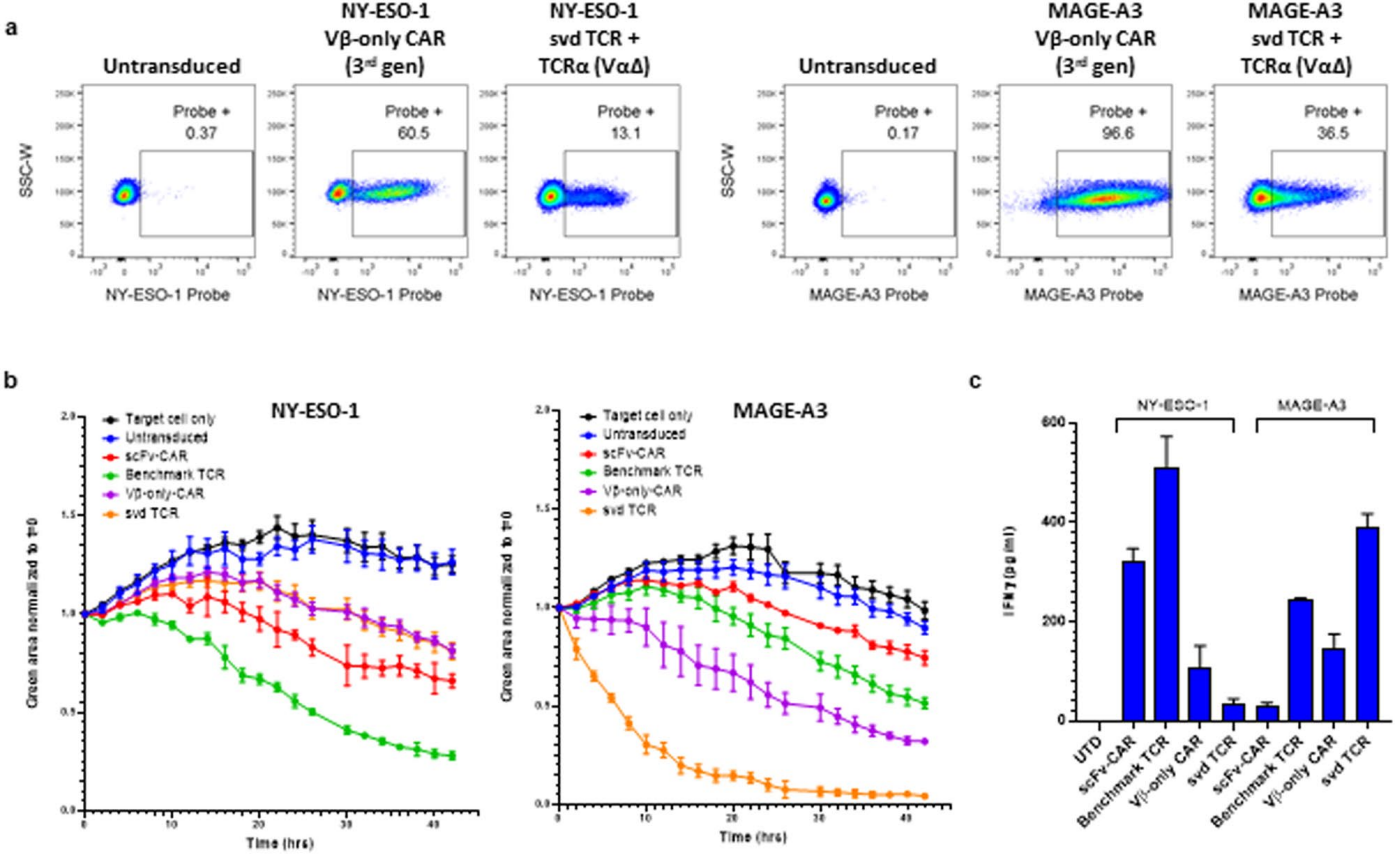
Vβ-only-CARs and svd TCRs expressed in primary T-cells show cytotoxicity and release cytokines. (**a**) Primary T cells transduced with indicated constructs stained with NY-ESO-1 or MAGE-A3 probes. (**b**) A375 cells expressing nuclear locating GFP loaded with 10 μM NY-ESO-1 (left) or MAGE-A3 (right) peptides were co-cultured with T cells transduced with NY-ESO-1 (left) or MAGE-A3 (right) binding constructs at 1:1 ratio and imaged in IncuCyte for 42 hours. Ratio of total green fluorescent area at each time point divided by time zero value is plotted. The error bar indicates SD (n = 2). (**c**) IFNγ measured by CBA assay with supernatants from the 24 hour time-point of the co-cultures in (**b**). The error bars indicate SD (n = 2).

To evaluate the effect of Vβ-only domain constructs on T cell activity, primary T cells were transduced with lentivirus and expression was confirmed by NY-ESO-1 or MAGE-A3 tetramer staining (Fig. 6a). CAR constructs expressed much better than TCR constructs, most likely due to the mispairing of the introduced TCR chains to endogenous TCR chains. Transduced T cells were used in an IncuCyte cell killing assay that enables visualization of target and effector cells by microscopy at 37 °C over time. A375 cells that stably express nuclear locating GFP were loaded with 10 μM NY-ESO-1 or MAGE-A3 peptides and co-cultured with transduced T cells at 1:1 ratios. T cell number was adjusted according to the transduction percentage measured by tetramer staining.

For NY-ESO-1 binders, T cells expressing the benchmark TCR showed the most potent cytotoxic activity, followed by T cells expressing the scFv-CAR, and the Vβ-only domain constructs in CAR and TCR formats which had similar killing activities (Fig. 6b). IFNγ measured in the supernatant of the co-culture at 24 hours showed a similar trend (Fig. 6c). K562 cells that overexpress single chain NY-ESO-1-β_2_m-HLA-A2 trimer^27^ and GFP were also used as target cells in the real-time killing assay. In this situation where the antigen is presented abundantly, all 4 NY-ESO-1-targeted constructs showed similar killing activities (Supplementary Fig. S8).

T cells expressing the MAGE-A3 benchmark TCR and scFv-CARs only showed mild cytotoxic activities while the Vβ-only-CAR and svd TCR triggered more robust killing (Fig. 6b). However, these Vβ-only-CAR and svd TCR cells also showed weak cytotoxicity toward K562 cells without any MAGE-A3 peptide (Supplementary Fig. S8), suggesting that these constructs likely trigger ligand-independent apoptosis of target cells. This is consistent with the high background NFAT signal observed in Jurkat cells transfected with the MAGE-A3 Vβ-only-CAR and svd TCR (Fig. 4c).

## Discussion

We have created Vβ-only domains that express, specifically recognize cognate pMHC ligands, and function robustly in Jurkat and primary T cells. The generality of this effect is suggested by the isolation and characterization of multiple binders against two different pMHC targets.

In a TCR format, the β chain utilize a surrogate α chain that lacks a Vα segment and forms activation-competent TCRs complexed with the six CD3 subunits. We presume the requirement for a surrogate α subunit relates to the formation of a complex in the membrane with other CD3 subunits that comprise a functional TCR. Sequential N-terminal deletions demonstrate that the globular Cα domain is necessary for full activity of the svd TCR. In contrast, the presence of a Vα domain on the α subunit, opposite the single Vβ polypeptide, has no impact on surface expression, but interferes with ligand-binding, presumably via steric hindrance of the pMHC ligand to its cognate Vβ binding site.

Selectivity of the svd TCRs compares favorably with other TCRs and antibodies that bind pMHCs. They retain the HLA restriction for which they were selected. Thus, the fact that evolution has favored a two-domain ligand-binding structure does not necessarily preclude the creation of selective single-domain binders, a feature that also applies to the antibody gene family^28^. Rather, it may be that a repertoire composed of two chains generates necessary diversity through the agency of combinatorial association.

In addition, the ligand-binding module can be grafted onto CAR signaling constructs where it functions independent of a TCR α chain. Both ligand-binding domains examined most closely (one NY-ESO-1 and one MAGE-A3 binder) are compatible with a variety of CAR formats that incorporate different CAR components (Figs. 1,3). Interestingly, the CAR versions of the Vβ-only ligand-binding domains have similar sensitivity to their TCR cousins. This result suggests that the properties of the ligand-binding domain determine functional sensitivity, regardless of the format; i.e., it is not the detailed structure of the signaling components, but rather, the binding properties of the receptor that dictate sensitivity, at least in the Jurkat NFAT-activation assay.

This conclusion is strengthened by the observation that scFv sequences derived from antibodies generated against the same pMHC targets (NY-ESO-1 and MAGE-A3), when grafted onto TCR subunits, yield hybrid TCRs that are nearly identical in sensitivity to CAR constructs that harbor the same scFvs (Supplementary Fig. S6). Thus, with two different pMHC targets and two independent origins of ligand-binding domains, one pair derived from TCRs and the other from antibodies, the sensitivity uniformly tracks with the ligand-binding domain, not the type of signaling molecule. These results again suggest that TCRs may acquire their striking sensitivity not by their highly evolved, multi-subunit mechanism, but rather through the process of selection in the body. It is possible that both α and β chains need to interact with the cognate MHC to deliver a sensitive signal. However, analysis of previously reported TCR-pMHC structures and amino acid sequences suggest that there are multiple instances where CDR3α makes no contact with the target pMHC whereas CDR3β contact is always present^29^. For TCRs isolated and studied *in vitro* by investigators, their extraordinary potency may be further explained by additional functional selection through growth on antigen-positive substrates or picking rare antigen-responsive T cells clones among hundreds of thousands of T cells^16,17^.

Our observations run contrary to the conclusion of Harris *et al*. who engineered two human TCR Vα/β pairs directed at either a WT-1 or MART-1 pMHC^30^. When these affinity-matured binders were tested as either reconstructed TCRs or CARs, the TCR versions were 10–100x more sensitive in both cases. The reasons for the discrepancy remain to be established, but include the possibilities that: (i) The Vα/β interaction is a critical part of the TCR signaling mechanism, and therefore the Vβ-only TCR is crippled with respect to TCR sensitivity because it lacks Vα; (ii) IL-2 expression used by Harris *et al*. as a readout favors the TCR signaling mechanism compared to NFAT activation measured in the Jurkat assay conducted by us; (iii) their use of a murine T cell hybridoma line and murine primary T cells as effectors with a human T2 lymphoid line as stimulus creates an interspecies activation scenario that somehow biases sensitivity toward TCRs; or, (iii) optimal TCR function follows from TCRs that are subjected to strong functional selection; if they originate from physical binding selections like the ones studied here, they lack the extraordinary sensitivity characteristic of some functionally-selected TCRs and, instead, display sensitivities equivalent to their CAR counterpart.

Signaling behavior of the Vβ-only CARs and svd TCRs are similar in many respect to TCRs regarding the shape of the dose/response curve (Fig. 4). However, they are less sensitive than the most highly selected or optimized TCRs, which are typically obtained from patients’ functionally selected T cells, or otherwise optimized for activity^16,17^, including the benchmark NY-ESO-1 TCR used in our experiments. The MAGE-A3-targeting Vβ-only module showed high background (tonic signal) and a narrow activation window in Jurkat cells (Figs. 3, 4c). Note that Vβ-only modules used in this study are not optimized. Sensitivity, tonic signaling, and activation windows are properties that can be optimized by screening larger numbers of variants (unpublished data). For example, the other three idiotypes (ID #1, 2, 4) of MAGE-A3 Vβ-only modules show much lower tonic signal than ID #5 (Supplementary Fig. S3), which we initially chose for further study due to its strongest tetramer binding ability (Supplementary Fig. S1). Based on a broad range of studies with TCRs, we believe efforts to optimize binding of Vβ-only-modules would produce more potent receptors^31–35^. In other words, there is nothing obvious in a Vβ-only ligand binding domain that precludes it from mimicking a natural TCR in all its fundamental attributes. However, we acknowledge that further validation and optimization will be needed to prove the utility of this technology beyond its academic interest.

Considerable effort has been directed at antibodies to engineer single-variable domains that avoid the requirement for a second chain. The goal is to make the ligand-binding function smaller and more easily shuttled from one molecular setting to another. The existence of natural single-domain antibodies, notably from the family Camelidae (including camels and llamas) and class Chondrichthyes (including sharks), has pointed the way toward engineering similar fully human domains. These small phylogenetically distant single-domain antibodies represent a significant variation of the classic immunoglobulin fold in which the surface that is typically involved in the interaction with a light chain variable domain is altered and covered by an extension of the CDR3 loop, resulting in a more compact, convex globular structure^36–38^. Efforts to engineer antibody structures to produce single-domain (e.g., heavy-chain only) variable domains, have required the introduction of substantial amino acid variation and structural change that create molecules more similar to the camelid single-domain molecules, compared to the two-chain structures from which they derive^39,40^.

Although we have not elucidated the atomic structure of the Vβ-only domains described here, we strongly suspect that they also involve considerable deviation from the two-chain TCR. Notably, all the Vβ-only domains identified use the Vβ5–8 variable gene segment. This bias is not explained by a skewed TCR input library because TRB-5–8, the V gene segment involved, was present at roughly the expected frequency in the HuTARG^TM^ library prior to induced TCR rearrangement (~1% of total V segments; data not shown). Its frequency was increased about 6-fold among cells that express surface TCRs, consistent with the view that it may more readily form stable globular domains. This possibility is supported by modeling (Fig. 2).

Vβ-only domains provide an opportunity to create bifunctional TCR ligand-binding domains that are smaller and more portable—potentially very useful in the context of CAR-T and TCR-T engineering. In particular, investigators have created bifunctional CARs that incorporate scFvs in tandem to direct engineered T cells at two separate antigens; e.g., CD19 and CD20^23,24^. The hypothesis is that such dual-targeted receptors will pose additional obstacles for tumor relapse, as the tumor must escape two rather than one T-cell targeting mechanisms. Such approaches have also been proposed for CARs, but the technical route toward TCR bifunctionality is less clear.

We have taken a significant step toward this goal by demonstrating that Vβ-only domains can be linked in tandem with only modest decline in their individual sensitivities to generate small, bifunctional single-Vβ-based CARs and TCRs. In principle, deployment of suitable TCR Vβs directed at different pMHCs permits construction of hybrid molecules activated by both pMHCs in a single engineered T cell, without either the problems associated with mispairing of multiple transferred subunits or the size limits imposed by current viral gene transfer vectors.

Interestingly, the bifunctional TCRs display improved functional selectivity compared to the individual binding domains tested on their own (Fig. 5b,d). Particularly the MAGE-A3 svd TCR exhibits some evidence of off-target activation when exposed to HLA-A2-positive T2 cells. However, in the bifunctional format this background nearly disappears. Though we do not have a simple explanation for this change, it suggests that the bifunctional format is at least compatible with production of stable, well-behaved surface receptors that bind ligands specifically.

## Methods

### Cell culture

HEK-293T (ATCC CRL-3216™), Jurkat clone E6-1 (ATCC TIB-152™), T2 (ATCC CRL-1992™), SUP-T1 (ATCC CRL-1942™), A375 (ATCC CLR-1619™) and K562 (ATCC CCL-243™) cell lines were used in this study. HEK-293T and A375 cells were maintained in DMEM supplemented with 10% fetal bovine serum (FBS) and 1% penicillin/streptomycin. Jurkat and SUP-T1 cells were maintained in RPMI supplemented with 10% heat-inactivated FBS and 1% penicillin/streptomycin. T2 cells were maintained in IMDM supplemented with 20% FBS and 1% penicillin/streptomycin. K562 cells engineered to stably express single chain NY-ESO-1-β_2_m-HLA-A2 trimer^27^ and GFP were generated and maintained in RPMI supplemented with 10% heat-inactivated FBS and 1% penicillin/streptomycin. Cells were incubated at 37 °C in 5% CO_2_.

### Plasmid construction

All constructs, except for the recovered Vβ-only pool from the HuTARG^TM^ platform (Patent WO2017/091905), were generated by Golden Gate assembly into the pLenti backbone, which has an EF1α promoter. NY-ESO-1 and MAGE-A3 scFvs on the control CARs were obtained from binder screens using the HuTARG^TM^ platform. Sequences for the control NY-ESO-1 and MAGE-A3 TCRs were obtained from patents US8143376B2 and W02012054825A1, respectively.

### HuTARG^TM^

See patent WO2017/091905 for details. Briefly, HEK293.2sus (ATTC CRL15733™) cells were engineered to contain a library of all combinations of unrearranged V, D and J segments of the β chain TCR locus. Because the host cells are constructed to contain a single loxP integration site, each cell post transfection of the library contains at most a single integrated copy of one of the roughly 1,800 combinations of V-D-J (64 × 2 × 14) gene segments, each segment is flanked by a recombination signal sequence (RSS). When triggered to rearrange by addition of an exogenous inducer that activates RAG1 expression, the RSSs mediate intramolecular site-specific recombination such that the population of cells produce a large repertoire of TCRβ chains that are further subjected to drug selection for in-frame recombinants; the TCRβ chain is expressed as a fusion with the puromycin resistance gene. The cells also contain a surrogate single alpha chain for pairing and surface expression.

### Transfection

Transient transfections of T-cell lines were performed using the Neon Transfection System (Thermo Fisher Scientific, Cat. No. MPK5000) according to the manufacturer’s instructions. Cells were pulsed three times at 1,500 V and a width of 30 msec. Transient transfection of HEK-293T cells were performed using the Fugene HD transfection method (Promega, Cat. No. E2311) according to the manufacturer’s instructions.

### FACS analysis

18 hours post-transfection, cells were harvested and washed three times with FACS buffer (PBS + 0.1% BSA). The cells were incubated with TCR βF1 (8A3) antibody conjugated with PE-Cy7 (Life Technologies, Cat. No. 25576641), CD3 epsilon antibody conjugated with FITC, Alexa Fluor 647-Streptavidin (Jackson, Cat. No. 016-600-084)-labeled MHC Class I A*02:01 SLLMWITQV (NY-ESO), or Alexa Fluor 647-Streptavidin-labeled MHC Class I A*02:01 FLWGPRALV (MAGE-A3) for 1 hour at 4 °C. Cells were then washed twice, stained with DAPI or the near-IR dead cell stain kit reagents (Thermo Fisher, Cat. No. L34976), and resuspended in FACS buffer for flow cytometer analysis. Data acquisition used the BD Canto instrument and software. Data were analyzed using Flowjo software.

### Homology model and hydrophobic patch calculation

3D structural models were generated in Prime homology modeling suite (Schrödinger, LLC) using the crystal structure of the MAGE-A3 TCR (TRAV21/TRBV5-1 family; PDBID: 5BRZ) as the template. All structures were prepared using the Schrödinger Protein Preparation Wizard^41^ before further calculations.

Hydrophobic patches on the surface of the homology model and crystal structures were calculated and visualized using the Protein Surface Analysis tool as implemented in Bioluminate (Schrödinger, LLC)^42^.

### Molecular dynamics simulation

The system was solvated using the Desmond System Builder (Schrödinger, LLC). Sodium ions were added to neutralize the system. The OPLS3e force field was utilized for all calculations^43^. The system equilibrated using the relaxation protocol as implemented in the Schrödinger package. Molecular Dynamics simulations were performed in the NPT ensemble at 300 K and 1 atm pressure using Desmond (D.E. Shaw Research). The reversible reference system propagation algorithm (RESPA) multiple time step approach was used with a time step of 2 psec and long-ranged electrostatic interactions were computed every 6 psec.^44^. Van der Waals and short-range electrostatic interactions were cut off at 9 A° and smooth particle mesh Ewald (PME) method^45^ was employed for calculation of long range electrostatic interactions. The temperature was controlled using a Nose-Hoover chain thermostat^46^ and the pressure was controlled using the Martyna-Tobias-Klein barostat^47^. Three simulations of 100 ns each were performed using the last frame as the initial coordinates on a Quadro P5000 GPU card with coordinates saved every 1.2 ps for subsequent analysis.

### NFAT Luciferase assay

NY-ESO-1 peptide (SLLMWITQV), a variant of the native peptide where cysteine at position 9 is mutated to Valine^48^, and MAGE-A3 peptide (FLWGPRALV) were synthesized by Genscript (Piscataway, NJ). Target peptides were serially diluted 3-fold starting at 100 μM and loaded onto 10,000 T2 cells in RPMI plus 1% BSA and 0.1% penicillin/streptomycin. Eighteen hours later, 12,000 transfected Jurkat cells encoding a NFAT-luciferase reporter were resuspended in RPMI/10% FBS/0.1% penicillin/streptomycin and added to the peptide-loaded T2 cells. The co-culture was incubated for 6 hours at 37 °C. One-step luciferase assay system (BPS Cat. No. 60690) was used according to manufacturer’s instructions to read luminescence on a microplate luminometer at 100 ms. Each experiment was done in duplicate.

### Primary T-cell transduction and activation

Peripheral blood mononuclear cells (PBMCs) and lentivirus were obtained from Alstem. PBMCs were activated with human T-cell TransAct™ according to manufacturer’s instructions in X-vivo media. For the transduction, lentivirus was added directly to the cells at a MOI of 10. Transduced primary T-cells were expanded in X-vivo medium with 1% human serum, 0.1% penicillin/streptomycin, 10 ng/ml IL-15, and 10 ng/ml IL-21. Cells were quiesced for 48 hours without stimulation or cytokines prior to assays.

### Cytotoxicity and IFNγ assay

A375 cells were transfected with nuclear locating GFP (IncuCyte NucLight Green) and selected for stable integration. Five thousand A375 cells were seeded with 10 μM of NY-ESO-1 or MAGE-A3 peptides. After 18 hours, cells were treated with 10 μg/ml of mitomycin C for 1 hour and washed three times. Five thousand transduced T-cells (adjusted for transduction percentage measured by tetramer staining) were added and monitored using the IncuCyte S3 live-cell analysis system (Essen Bioscience) at 37 °C and 5% CO_2_. Images were captured every two hours for 42 hours using a 10X objective. Green area at each time point was normalized to time zero to measure loss of live target cells. Experiments were done in duplicate.

Supernatants were harvested at the 24 hour time-point of the cytotoxicity assay co-culture. IFNγ levels were measured using the BD Cytometric bead array system according to manufacturer’s instruction.

### Statistical analysis

For all data comparisons, the Student’s t test was performed using GraphPad Prism software. EC_50_ values were obtained by fitting the peptide concentration-dependent NFAT-Luciferase readout by nonlinear regression.

## Supplementary information

Supplementary Information
