# Supplementary material for: Single variable domains from the T cell receptor β chain function as mono- and bifunctional CARs and TCRs
Source: Sci Rep. 2019 Nov 21;9:17291. doi: 10.1038/s41598-019-53756-4 (PMC6872726; doi:10.1038/s41598-019-53756-4)
Supplement: Supplementary file 1 — Supplementary Information [file 41598_2019_53756_MOESM1_ESM.pdf]

Supplementary Information

Single variable domains from the T cell receptor β chain function as mono- and bifunctional CARs and TCRs

Julyun Oh[1], Dora Toledo Warshaviak[1], Mikayel Mkrtichyan[1], Melanie Munguia[1], Abby Lin[2], Falene Chai[2], Craig Pigott[2], Jaspal Kang[2], Michael Gallo[2], Alexander Kamb[1*]

Supplementary Figure S1. Expression and target pMHC tetramer binding of Vβ domains

FACS plot of HEK293T cells transfected with Vb-only domains recovered from the HuTARG™ system. The x-axis reflects the amount of target pMHC probes bound to cell. The y-axis reflects the amount of the TCRβ chain on the cell surface.

Supplementary Figure S2. Constructs show target specific NFAT activation

NFAT-luciferase signal of transfected Jurkat cells after 6hrs of co-culture with NY-ESO-1 (blue), MAGE-A3 (red), or unrelated (green) peptides-loaded T2 cells. The error bars indicate SD (n=2).

Supplementary Figure S3. Idiotypes of MAGE-A3 Vβ-only binders show a range of background signal.

NFAT-luciferase signal of transfected Jurkat cells after 6hrs of co-culture with MAGE-A peptide-loaded T2 cells. The error bars indicate SD (n=2).

Supplementary Figure S4. Fluorescence microscopy of stained constructs

Transfected Jurkats cells were fixed and stained with cognate target pMHC tetramers conjugated with PE in the presence or absence of nonionic detergent for permeabilization. The cells were then analyzed with a 40x objective for cellular localization of CAR constructs.

Supplementary Figure S5. Vb-only CARs with CD8 hinge

a, Schematics of the Vβ-only-CARs tested. **b,** NFAT-luciferase signal of transfected Jurkat cells after 6hrs of co-culture with NY-ESO-1 (left) and MAGE-A3 (right) peptide-loaded T2 cells. The error bars indicate SD (n=2).

Supplementary Figure S6. Alpha chain truncation

a, Surface expression of CD3ε and target probe binding in SUP-T1 cells measured by FACS staining. **b,** NFAT-luciferase signal of transfected Jurkat cells after 6hrs of co-culture with NY-ESO-1 peptide-loaded T2 cells. The error bars indicate SD (n=2).

Supplementary Figure S7. scFv-TCR fusion follows scFv-CAR sensitivity

a, Schematics of the constructs tested. **b,** NFAT-luciferase signal of transfected Jurkat cells after 6hrs of co-culture with NY-ESO-1 peptide-loaded T2 cells. The error bars indicate SD (n=2).

Supplementary Figure S8. Real-time killing assay with K562 target cells

Related to Fig 6B. K562 cells overexpressing single chain NY-ESO-1-β_2m-HLA-A2 trimer and GFP were co-cultured with T cells expressing NY-ESO-1 (left) or MAGE-A3 (right) targeted constructs at 1:1 ratios. Images were taken for 42 hours by IncuCyte. Total green fluorescent area at each time point divided by the t=0 value are plotted. The error bars indicate SD (n=2).

Supplementary Fig. S1

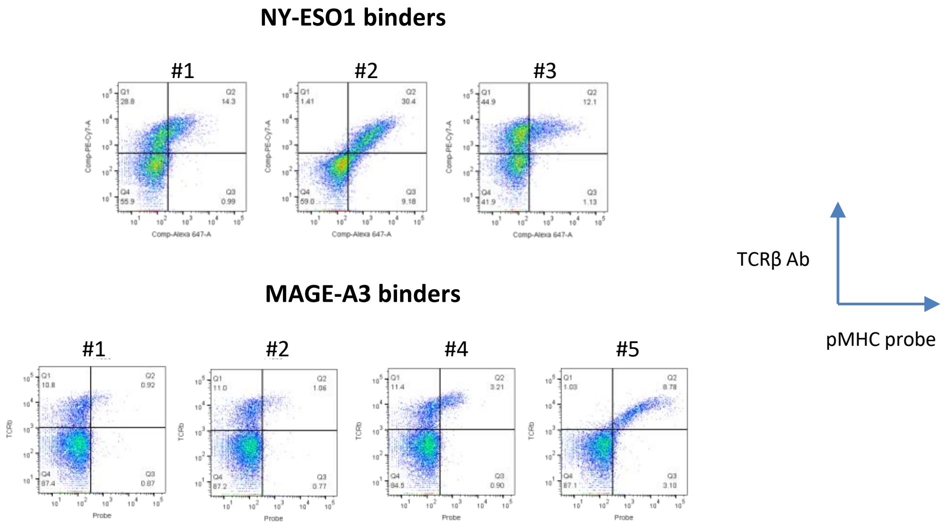

Supplementary Fig. S2

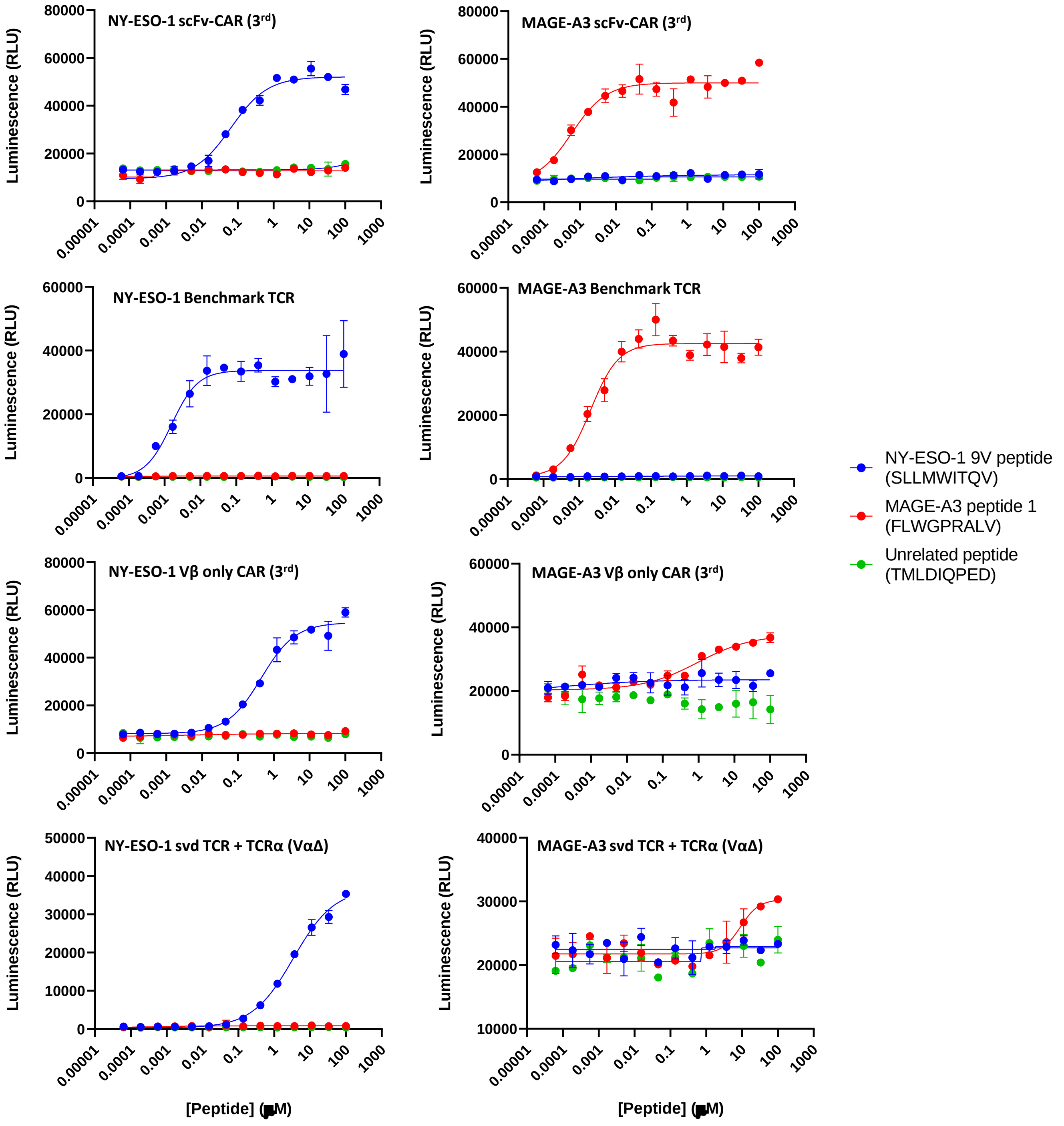

Supplementary Fig. S3

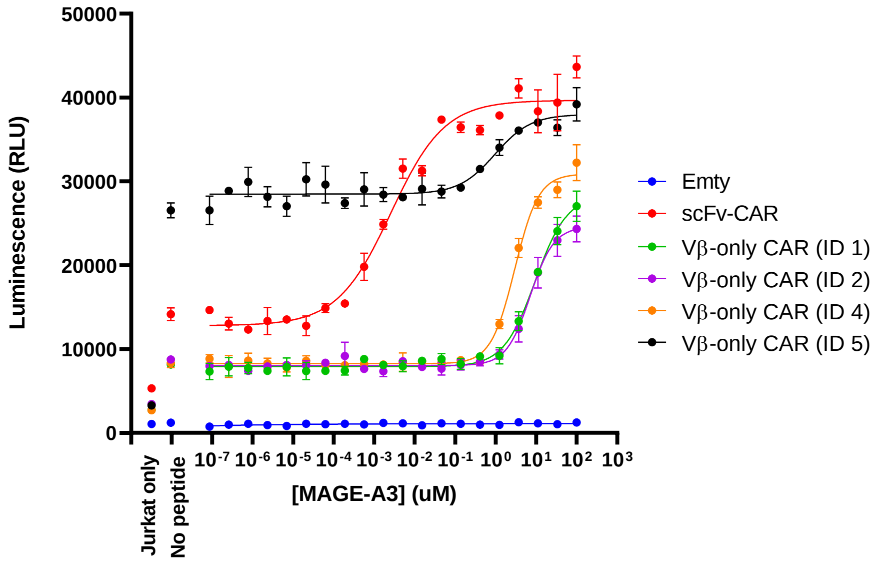

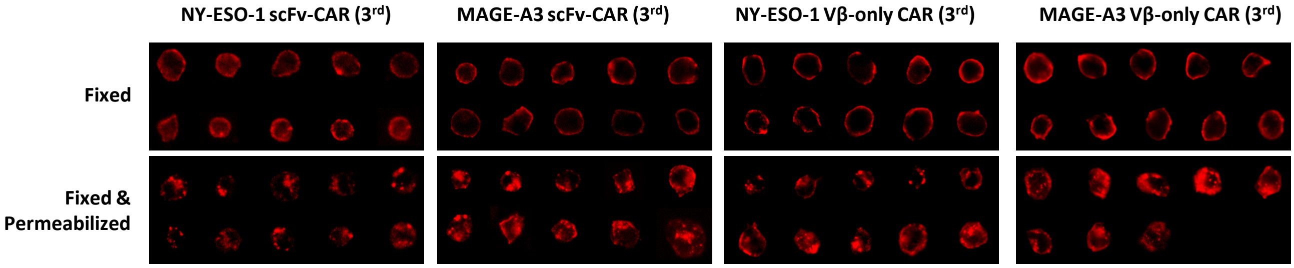
NY-ESO-1 scFv-CAR (3rd)
MAGE-A3 scFv-CAR (3rd)
NY-ESO-1 Vβ-only CAR (3rd)
MAGE-A3 Vβ-only CAR (3rd)
Fixed
Fixed &
Permeabilized

Supplementary Fig. S5

a

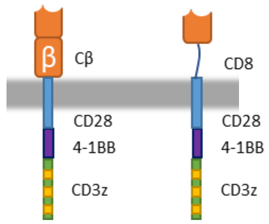

b

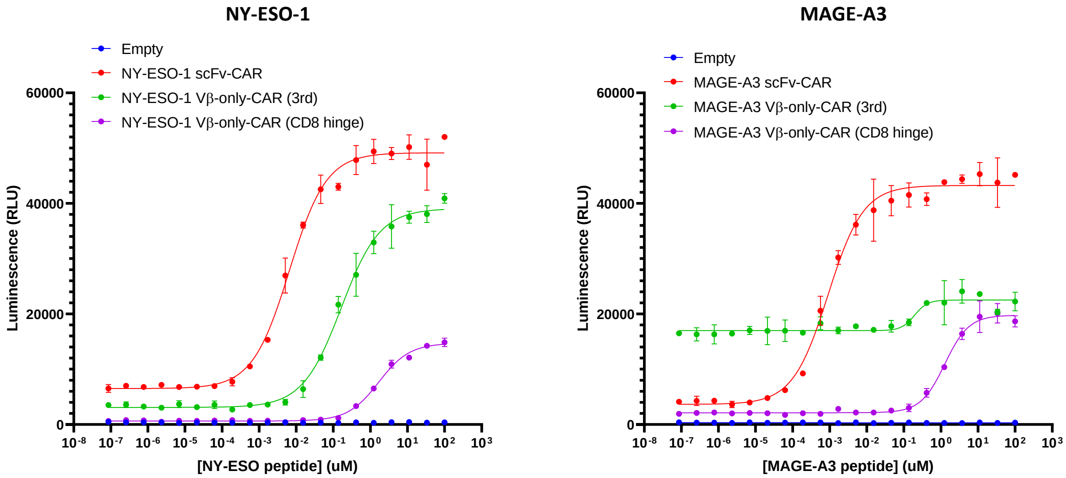

a

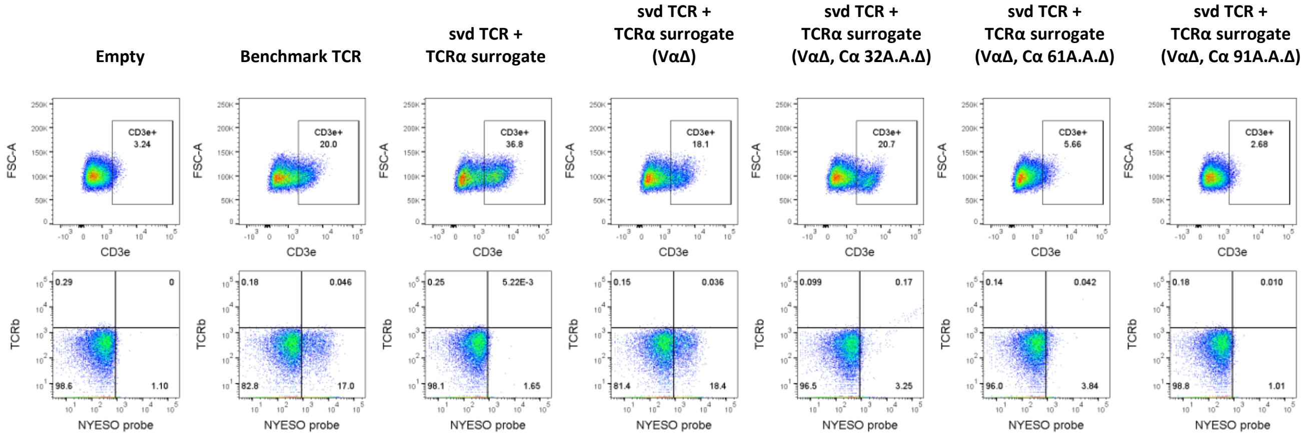
Empty
Benchmark TCR
svd TCR +
TCRα surrogate
svd TCR +
TCRα surrogate
(VαΔ)
svd TCR +
TCRα surrogate
(VαΔ, Cα 32A.A.Δ)
svd TCR +
TCRα surrogate
(VαΔ, Cα 61A.A.Δ)
svd TCR +
TCRα surrogate
(VαΔ, Cα 91A.A.Δ)
FSC-A
CD3e
CD3e+
3.24
20.0
36.8
18.1
20.7
5.66
2.68
TCRb
NYESO probe
0.29
0
98.6
1.10
0.18
0.046
82.8
17.0
0.25
5.22E-3
98.1
1.65
0.15
0.036
81.4
18.4
0.099
0.17
96.5
3.25
0.14
0.042
96.0
3.84
0.18
0.010
98.8
1.01

b

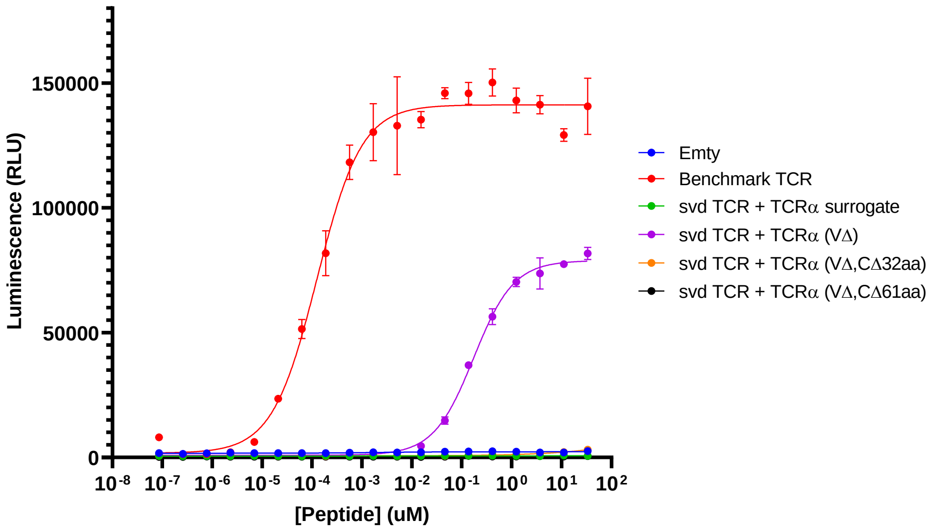
Luminescence (RLU)
150000
100000
50000
0
[Peptide] (uM)
Emty
Benchmark TCR
svd TCR + TCRα surrogate
svd TCR + TCRα (VΔ)
svd TCR + TCRα (VΔ,CΔ32aa)
svd TCR + TCRα (VΔ,CΔ61aa)

Supplementary Fig. S7

a

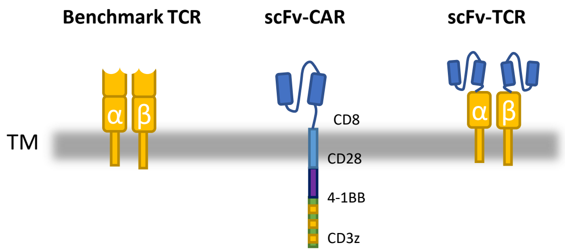

b

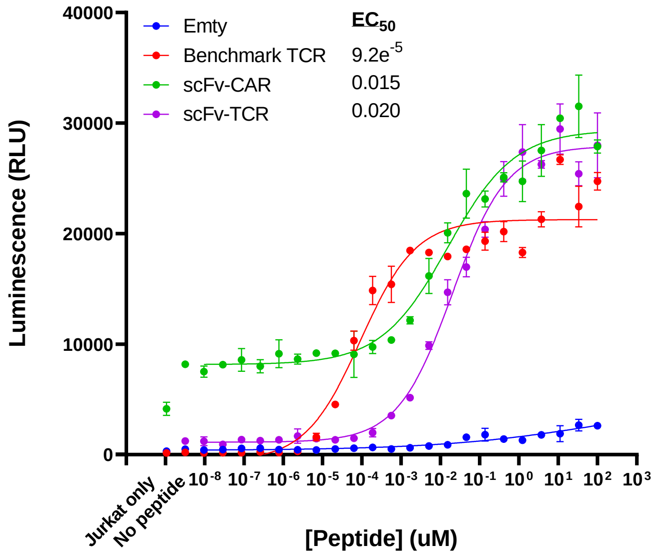

Supplementary Fig. S8

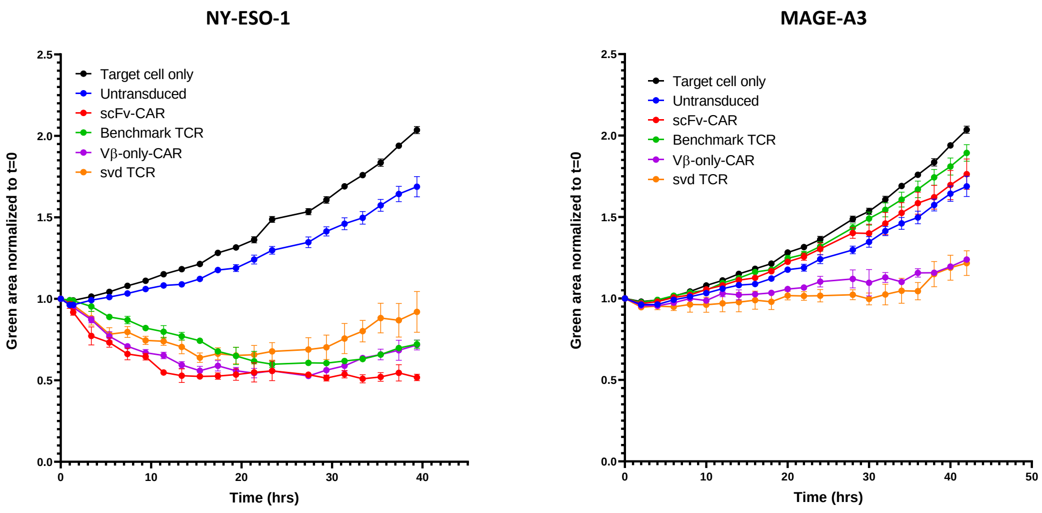

Supplementary Table 1. CDR3 sequence alignments of identified Vβ domains

Binder	ID#	TRBV Gene	TRBD Gene	TRBJ Gene	CDR3 Nucleotide Sequence	CDR3 Amino Acid Sequence
NY-ESO	1	TRBV5-8*01	N/A	TRBJ2-7*01	TGTGCCAGCAGCTTGGGCCTCGGCTACGAGCAGTACTTC	CASSLGLGYEQYF
NY-ESO	2	TRBV5-8*01	TRBD2*02	TRBJ2-1*01	TGTGCCAGCAGCTTGGGGGGTCCCCGGGGACTAGCGGGACTTAGGGGGGATGAGCAGTTC	CASSLGGPRGLAGLRGDEQF
NY-ESO	3	TRBV5-8*01	TRBD2*01	TRBJ2-1*01	TGTGCCAGCAGCTTAAGGCGGGACAATGAGCAGTTC	CASSLRRDNEQF
MAGE-A3	1	TRBV5-8*01	TRBD1*01	TRBJ2-3*01	TGTGCCAGCAGCTTGGAAGTCCTGCTGGGGGCGGATTTTCCAGATACGCAGTATTTT	CASSLEVLLGADFPDTQYF
MAGE-A3	2	TRBV5-8*01	TRBD2*02	TRBJ2-1*01	TGTGCCAGCAGCTTCCCGGCGGGACACGGGGCGGATCTGGACAATGAGCAGTTC	CASSFPAGHGADLDNEQF
MAGE-A3	4	TRBV5-8*01	TRBD1*01	TRBJ2-1*01	TGTGCCAGCAGCGAGATAACGGGGCGGATAGGGGAGCAGTTC	CASSEITGRIGEQF
MAGE-A3	5	TRBV5-8*01	TRBD1*01	TRBJ2-1*01	TGTGCCAGCAGCTTGGGGGGGGACGAGCTGGGGGCGGATGGGAATGAGCAGTTC	CASSLGGDELGADGNEQF

N/A = unassignable TRBD
BLUE: TRBV Sequence
BLACK: TRBD Sequence
PURPLE: TRBJ Sequence
RED: Product of terminal deoxynucleotidyl transferase (TDT)

Supplementary Table 2. EC_{50} from NFAT Luciferase assay

Figures	Constructs	EC_{50} (mM)	
		NY-ESO-1 binders	MAGE-A3 binders
Fig. 3	Benchmark TCR	0.00006	0.002
Fig. 3	scFv-CAR	0.002	0.001
Fig. 3	svd TCR + TCRα surrogate	-	-
Fig. 3	Vβ-only CAR (CD3z)	0.210	1.2
Fig. 3	Vβ-only CAR (CD28-CD3z)	0.091	0.55
Fig. 3	Vβ-only CAR (CD28-41BB-CD3z)	0.100	0.99
Fig. 4C	scFv-CAR	0.008	0.001
Fig. 4C	svd TCR + TCRα surrogate	-	-
Fig. 4C	Vβ-only CAR (CD3z)	0.40	1.2
Fig. 4C	Vβ-only CAR (CD28-CD3z)	0.18	0.55
Fig. 4C	Vβ-only CAR (CD28-41BB-CD3z)	0.15	0.99
Fig. 4C	svd TCR + TCRα (VΔ)	0.83	2.7
Fig. 5B	Vβ-only CAR (CD28-41BB-CD3z)	0.16	0.19
Fig. 5B	N-M-Vβ-only CAR	3.0	0.28
Fig. 5B	M-N-Vβ-only CAR	0.37	1.8
Fig. 5D	svd TCR + TCRα (VΔ)	0.24	13
Fig. 5D	N-M-svd TCR + TCRα (VΔ)	0.21	4.7
Fig. 5D	M-N-svd TCR + TCRα (VΔ)	1.6	16
